# FsCGBP, a Cutinase G-Box Binding Protein, Regulates the Growth, Development, and Virulence of *Fusarium sacchari,* the Pathogen of Sugarcane Pokkah Boeng Disease

**DOI:** 10.3390/jof10040246

**Published:** 2024-03-25

**Authors:** Haoming Liang, Fang Li, Yundan Huang, Quan Yu, Zhenxin Huang, Quan Zeng, Baoshan Chen, Jiaorong Meng

**Affiliations:** 1 State Key Laboratory for Conservation and Utilization of Subtropical Agro-Bioresources, Nanning 530004, China; 2017392018@st.gxu.edu.cn (H.L.); 2217304009@st.gxu.edu.cn (F.L.); 2117304009@st.gxu.edu.cn (Y.H.); 2017304028@st.gxu.edu.cn (Q.Y.); 2017304007@st.gxu.edu.cn (Z.H.); 1908401002@st.gxu.edu.cn (Q.Z.); 19850001@gxu.edu.cn (B.C.); 2 Guangxi Key Laboratory of Sugarcane Biology, College of Agriculture, Guangxi University, Nanning 530004, China

**Keywords:** *Fusarium sacchari*, C2H2 transcription factor, FsCGBP, virulence, MAPK pathway, sugarcane Pokkah boeng

## Abstract

*Fusarium sacchari* is a causal agent of sugarcane Pokkah boeng, an important fungal disease that causes a considerable reduction in yield and sugar content in susceptible varieties of sugarcane worldwide. Despite its importance, the fungal factors that regulate the virulence of this pathogen remain largely unknown. In our previous study, mapping of an insertional mutant defect in virulence resulted in the identification of a cutinase G-box binding protein gene, designated *FsCGBP,* that encodes a C2H2-type transcription factor (TF). FsCGBP was shown to localize in the nuclei, and the transcript level of *FsCGBP* was significantly upregulated during the infection process or in response to abiotic stresses. Deletion or silencing of FsCGBP resulted in a reduction in mycelial growth, conidial production, and virulence and a delay in conidial germination in the *F*. *sacchari*. Cutinase genes *FsCUT2*, *FsCUT3*, and *FsCUT4* and the mitogen-activated protein kinase (MAPK) genes *FsHOG1, FsMGV1*, and *FsGPMK1,* which were significantly downregulated in Δ*FsCGBP*. Except for *FsHOG1*, all of these genes were found to be transcriptionally activated by FsCGBP using the yeast one-hybrid system in vitro. The deletion of individual cutinase genes did not result in any of the phenotypes exhibited in the Δ*FsCGBP* mutant, except for cutinase activity. However, disruption of the MAPK pathway upon deletion of *FsMGV1* or *FsGPMK1* resulted in phenotypes similar to those of the Δ*FsCGBP* mutant. The above results suggest that FsCGBP functions by regulating the MAPK pathway and cutinase genes, providing new insights into the mechanism of virulence regulation in *F. sacchari*.

## 1. Introduction

Pokkah boeng disease (PBD), first observed and characterized in Java, Indonesia, is one of the most devastating fungal diseases in most sugarcane production areas worldwide [1,2,3]. The term “Pokkah boeng” is a Javanese term that refers to a malformed or distorted top, which is the main PBD symptom. In the early stages of infection, the yellowing or chlorosis of young leaves is observed, with some leaves exhibiting red specks. As the symptoms develop, the leaves begin to crumple and twist. In the most severe cases, the top of the plant rots, which is termed top rot or stalk rot [1,4,5]. PBD can cause a significant reduction in sugarcane yield and sugar content [1,6]. In China, reductions of 30.2–48.5% in stalk yield and 2.63–5.21% in sugar content were reported for sugarcane infected with PBD, depending on the cultivars and environmental conditions [7].

PBD can be caused by several species of *Fusarium* within the *Fusarium fujikuroi* species complex, such as *F. sacchari*, *F. verticillioides*, *F. andiyazi*, and *F. subglutinans* in different countries [8,9,10,11]. Among these, *F. sacchari* is the dominant pathogen in most sugarcane production areas and is also associated with sugarcane wilting, a destructive fungal disease affecting production in many sugarcane growing regions [1,12,13]. *F. sacchari* was first reported to cause PBD in China in 2019 [11].

The pathogenesis of various *Fusarium* species, such as *F. graminearum* and *F. oxysporum*, has been studied extensively [14,15,16]. However, the pathogenic mechanism of *F. sacchari* remains largely unknown. In a recent study, the candidate secreted effector proteins (CSEPs) in the complete genome of *F. sacchari* were identified using bioinformatic approaches, and the functions of a few CSEPs, such as pectate lyase (FsPL), Nep1-like proteins (FsNLPs), and an effector protein that contains a carbohydrate-binding module domain designated Fs11724, were partially validated for their involvement in virulence [17,18,19,20].

C2H2-type transcription factors (TFs), the second largest family of fungal zinc finger regulators, are known to be associated with the regulation of development, stress tolerance, metabolic activities, and virulence in phytopathogenic fungi [21,22]. In *Aspergillus oryzae*, the C2H2-type TF *FlbC* regulates the transcription of *brlA*, *abaA*, and *vosA* and affects nutritional growth and conidiophore formation [23]. In the cotton pathogen *Verticillium dahliae*, a C2H2-type zinc finger TF, *V. dahliae* chorion TF 2 (VdCf2) plays key roles in growth, melanin production, and virulence [24]. PlCZF1, which contains a C2H2 zinc finger, was reported as a vital regulator for sexual development and virulence in *Peronophythora litchi* [25]. CfCpmd1, a C2H2 TF in *Colletotrichum fructicola*, is involved in plus and minus strain differentiation that also affects hyphal growth, sporulation, appressorium formation, and virulence [26]. The recently identified UvCGBP1 is a G-box binding protein in *Ustilaginoidea virens* and a C2H2 TF. It has been reported to affect pathogen growth and significantly attenuate virulence by regulating the mitogen-activated protein kinase (MAPK) pathway [27]. Upon searching the *F. sacchari* genome, a total of 54 C2H2-type TFs were predicted (our unpublished data). However, these TFs have yet to be functionally characterized.

In our previous study, a T-DNA insertional mutagenesis library containing 3801 *F. sacchari* transformants was constructed. A screening of the mutants using a detached leaf assay identified 32 virulence-impaired mutants [28]. One of these mutants, FsAT0345, was mapped to a gene encoding a cutinase G-box binding protein, designated as FsCGBP, which shares 54.0% identity and 65.7% similarity with the cutinase G-box binding protein, *UvCGBP1* in the rice pathogen *Ustilaginoidea virens.* In the current study, we showed that FsCGBP localizes in the nucleus in *F. sacchari* and FsCGBP-deletion mutants were defective in vegetative growth, conidial production and germination, and virulence. FsCGBP was capable of activating gene transcription for those encoding cutinase and MAPK-related proteins in vitro. In vivo characterization of the gene function showed that MAPK pathway disruption mimicked the defect phenotypes exhibited by the FsCGBP-deletion mutant, suggesting that FsCGBP exerts its functions mainly through MAPK pathway regulation. These results unveiled the critical role of FsCGBP in the growth, development, and virulence of *F. sacchari*.

## 2. Materials and Methods

### 2.1. Sugarcane Variety and Fungal Strains

PBD-susceptible sugarcane cultivar Zhongzhe 9 (ZZ9) was grown in the greenhouse, and plants at the 4–5 leaf stage were used for virulence assays. The *F. sacchari* wild-type (WT) strain FF001, which was isolated from Fusui, Guangxi Province, China [11], was used throughout the study. All fungal strains, including mutants used in this study, are listed in Table S1.

### 2.2. Fungal Culture Conditions

Fungal strains were initially grown on potato dextrose agar (PDA) (13.8 g/L) (Qingdao Hope Bio-Technology, Qingdao, China) at 28 °C in the dark for 6 days. For mycelium growth and conidiation assays, 4 mm diameter mycelial plugs of the WT strain FF001 and mutant strains were inoculated on PDA at 28 °C in the dark for 6 days. Conidia were harvested using sterile Miracloth (Calbiochem, San Diego, CA, USA), resuspended in sterile water, and quantitated using a hemocytometer. For the conidial germination assay, 100 μL of conidial suspension (1.0 × 10^6^ conidia/mL) was spread on an agar medium (2% agar) and incubated at 28 °C for 10 h. Germination was observed at 1 h intervals, and at least 100 spores per treatment were counted. For the stress response tests, 4 mm diameter mycelial plugs of each strain were, respectively, inoculated on CM plates (sucrose 10 g/L, yeast extract 6 g/L, tryptone 6 g/L) or CM plates supplemented with 1 M NaCl, 1 M sorbitol, 0.05% H_2_O_2_ (*v*/*v*), 0.3 mM Congo red (CR), or 0.5 mM sodium dodecyl sulfate (SDS) and incubated at 28 °C for 4 days. Each experiment was repeated three times.

For determination of the *FsCGBP* expression level in different germination stages, a 5 mL conidial suspension (1 × 10^8^ /mL) was inoculated into 150 mL of sugarcane leaf liquid medium (100 g of sugarcane leaves in 1 L of H_2_O, boiling for 1 h, cooled to room temperature, centrifuged at 200 rpm for 10 min). For determination of the *FsCGBP* expression level in different infection stages, conidial suspension (1 × 10^8^ /mL) was sprayed onto the surface of young sugarcane leaves, and the leaves were collected at times from 24 to 96 hpi, with an interval of 24 h. To compare *FsCGBP* expression under different abiotic stresses, 1 mL conidial suspension (1 × 10^6^ /mL) was added to 150 mL CM liquid medium or CM liquid medium supplemented with 1 M NaCl, 1 M sorbitol, 0.01% H_2_O_2_ (*v*/*v*), 0.3 mM CR, or 0.5 mM SDS, and incubated at 28 °C at 200 rpm for 2 days. To compare *FsCGBP* expression, *FsCUTs* genes, as well as the MAPK genes in the WT and mutant strains, 1 mL conidial suspension (1 × 10^6^ /mL) of each strain was individually added to 150 mL potato dextrose water (PDW) medium and incubated at 28 °C at 200 rpm for 2 days. The cultures of all strains were filtered, and the mycelium and spores were used for the subsequent quantitative real-time polymerase chain reaction (qRT-PCR) analysis.

### 2.3. Sequence Analysis

The sequences of *FsCGBP* and other genes of *F. sacchari* used in this study were obtained from the genome of the WT strain FF001 (our unpublished data). The relative molecular weight was predicted using the Compute pI/Mw tool (http://www.expasy.ch/tools/pi_tool.html). Protein structural domain analysis was conducted using the National Center of Biotechnology Information (NCBI) Conserved Domains (https://www.ncbi.nlm.nih.gov/Structure/cdd/wrpsb.cgi) and protein sequences. FsCGBP orthologs were identified using a search of the NCBI database with the FsCGBP protein sequence used as a query using the BLASTP tool (http://www.ncbi.nlm.nih.gov/BLAST/). Protein sequence consensus and identity analysis were performed using Vector NTI advance 11.5.1 (Invitrogen, Carlsbad, CA, USA). The protein sequence alignments and phylogenetic analysis were performed using MEGA 7.0 and the neighbor-joining algorithm with 1000 bootstrap replicates [29]. Protein-conserved structural domains were visualized and compared using Jalview 2.11.2.7.

### 2.4. Construction of the Plasmids

All plasmids involved in this study were constructed with the Basic Seamless Cloning and Assembly Kit (TransGen Biotech, Beijing, China). The resulting constructs were transformed into *Escherichia coli* (TransGen Biotech, Beijing, China) and confirmed using PCR and sequencing (Sangon Biotech, Shanghai, China). Plasmids and primers used are listed in Table S2 and Table S3, respectively.

### 2.5. Transactivation Activity Assays

The transcriptional activity of *FsCGBP* was measured with the *S. cerevisiae* strain Y2H Gold, as described by Su et al. [30]. The full *FsCGBP* coding region was cloned and inserted into the *Eco*RI and *Bam*HI sites of the pGBKT7 vector (Tarkara, Beijing, China) to generate the constructed pGBKT7-FsCGBP plasmid. The pGBKT7-*FsCGBP* plasmid was then transformed into Y2HGold competence cells, and the transformed strains were isolated with SD/-Trp medium and confirmed using PCR. The correct yeast suspension aliquots, diluted to 10^−1^, 10^−2^, and 10^−3^, and 10 μL of each dilutant, were separately incubated on plates containing SD/-Trp-His or SD/-Trp media supplemented with X-α-Gal (20 mg/mL) and allowed to grow at 28 °C for 3 days. pGBKT7 was used as a negative control. The transactivation activity was evaluated according to the growth status of colonies and the blue color manifested by X-α-gal activity.

### 2.6. Yeast One-Hybrid Assay

A yeast one-hybrid assay was performed as described by Reece and Marian, with a slight modification [31]. The FsCGBP coding sequence region was cloned and inserted into the pGADT7 vector (Takara Biomedical Technology, Beijing, China) at *EcoR*Ⅰ and *BamH*Ⅰ sites to generate the pGADT7-FsCGBP prey construct and subsequently transformed into Y1HGold cells (Takara Biomedical Technology, Beijing, China). An approximately 1000 base-pair sequence was extracted upstream of the target genes (*FsCUT1*, *FsCUT2*, *FsCUT3*, *FsHog1*, *FsMGV1*, or *FsGPMK1*) and the PlantCARE online tool (http://bioinformatics.psb.ugent.be/webtools/plantcare/html/) was used to predict the conserved G-box element in the promoter region. A sequence of 100–300 bp in the promoter region containing the G-box element was cloned into the pAbAi vector (Miaoling Biotechnology, Wuhan, China) at the *Sac* Ⅰ and *Sal* Ⅰ sites to create a series of pAbAi-pro bait constructs. Each bait construct was linearized by *Bst*BⅠ and separately transformed into pGADT7-*FsCGBP* competent cells. The transformed strains were screened on SD/-Leu-Ura medium and confirmed using PCR. The transformed yeast cells, along with the controls, were incubated on SD/-Leu-Ura media supplemented with 500 ng/mL aureobasidin A (AbA) at 28 °C for 3 days and evaluated for protein–DNA interactions based on growth ability. The yeast strains containing the p53-AbAi and p53-AbAi+pGADT7-p53 plasmids were used as the negative and positive controls, respectively.

### 2.7. Subcellular Localization

The enhanced green fluorescent protein (eGFP) gene sequence and full-length coding sequence of *FsCGBP* were cloned into the pCPXHY2 vector at the *Not*Ⅰ site in order to generate the pCPXHY2-eGFP-*FsCGBP* construct. The obtained construct was then transformed into the protoplasts of the WT strain FF001 using a polyethylene glycol (PEG)-mediated genetic transformation system and screened on a PDA medium containing 100 mg/mL hygromycin B. The nuclei of the conidia or hyphae were stained with 4′,6-diamidino-2-phenylindole (DAPI) (Invitrogen, CA, USA) at a final concentration of 10 μg/mL. Hyphae or conidia were viewed under an Olympus BX53 fluorescence microscope (Olympus, Tokyo, Japan).

### 2.8. Scanning Electron Microscope (SEM) Microscopy

A small piece of the fungal mycelium from the colony edge was collected, and the sample was prepared according to previously described methods [32]. The samples were viewed using a Hitachi H-7500 SEM (Hitachi, Tokyo, Japan).

### 2.9. Generation and Complementation of the Mutants

Total genomic DNA was extracted from mycelia as described in [33]. To generate the *FsCGBP* knockout mutants, approximately 1.5 kb of the upstream and downstream fragments of *FsCGBP* were amplified using the total genomic DNA as a template. The hygromycin-B-resistant gene coding region was flanked upstream (left) and downstream (right) using fusion PCR [34]. The resulting fusion fragment was used to transform the protoplasts of the WT strain. The resulting transformants were then screened on PDA containing hygromycin B (50 mg/L), and the positive transformants were further screened using PCR to identify the *FsCGBP*-deletion mutants (ΔFsCGBP) [35]. The deletion mutants of *FsCUT1*, *FsCUT2*, *FsCUT3*, *FsCUT4, FsHOG1*, *FsMGV1,* and *FsGPMK1* were constructed using the same strategy.

To generate the *FsCGBP* complementation strain of ΔFsCGBP (CΔFsCGBP-1), a fragment of approximately 4.6 kb, which contained the full *FsCGBP* coding region and its upstream and downstream sequences, was amplified using total genomic DNA as the template and cloned into the pCPXG418 vector at the *EcoR*Ⅰ and *Not*Ⅰ sites. The complementary construct was identified using PCR, confirmed by sequencing, and then transformed into the protoplasts of the ΔFsCGBP-1 strain. The transformants were screened on PDA with 50 mg/L geneticin and confirmed at the mRNA level using reverse transcriptase PCR (RT-PCR).

In order to obtain the RNAi-silencing strains, an intron fragment of 287 bp from the oxysterol-binding protein gene (our unpublished data) was selected. A fragment of 583 bp from the *FsCGBP* cDNA sequence (coordinates +247–+829) predicted f using the online software siDirect (sidirect2.rnai.jp/) was used as the siRNA target and amplified using *F. sacchari* cDNA as the template with gene-specific primers (Table S1). The silencing fragment with the intron flanked by the RNAi fragments in the opposite direction was inserted into the pCPXHY2 vector at the *Not*I and *Sph*I sites to generate the RNAi construct pCPXHY2-RNAi-*FsCGBP*. The resulting construct was then used to transform the protoplasts of the WT strain FF001. The transformants were screened on PDA with 50 mg/L hygromycin B and confirmed using PCR.

### 2.10. qRT-PCR Analysis

Total RNAs of the mycelia, conidium, or infected sugarcane leaves were extracted using the FastPure^®^ Universal Plant Total RNA Isolation Kit (Nanjing Vazyme, Nanjing, China) according to the manufacturer’s instructions. The cDNA templates were prepared with the HiScript^®^ Ⅲ 1st Strand cDNA Synthesis Kit and gDNA wiper (Vazyme, Nanjing, China). The qRT-PCR was performed with the ChamQ Universal SYBR qPCR Master Mix (Vazyme, Nanjing, China) using the Lightcycler^®^ 96 SW1.1 PCR system. Data from three biological replicates were collected and used for analysis. Fungal gene expression levels were quantified using *β-Actin* gene transcript as internal references, and the relative expression levels were calculated using the 2^−ΔΔCt^ method. The primers used for the qRT-PCR are listed in Table S1.

### 2.11. Virulence Assay

Virulence assays were performed with detached leaves or whole plants [2]. For the detached leaf assay, healthy and fresh sugarcane leaves were collected from sugarcane plants grown in the greenhouse at the 4–6 leaves stage and cut into 6 cm long pieces. A 1.0 cm wound was then made using scissors at the leaf margin and inoculated with a 4 mm diameter mycelial plug of *F. sacchari*, cultured on PDA for 6 days. The inoculated leaves were incubated in a growth chamber with 90–95% humidity at 28 °C. The photographs were taken at 4 dpi, and the lesion areas on the leaves were quantified and evaluated using Image J. Sterile PDA plugs were used as the negative control. The relative lesion area ratio (%) was calculated as (lesion area of deletion or silencing mutant/ lesion area of WT strain FF001) × 100. For whole plant inoculation, 300 uL of conidial spore suspension in H_2_O at 10^4^ conidia/mL was injected into the stalk apical tissue with the aid of a syringe [2]. Symptoms were recorded at 7 and 14 days post-inoculation. Disease severity was scored based on the disease severity index (DSI) [2]. To inspect the spread of fungal mycelia inside plants, sugarcane tissue samples were stained with trypan blue dye (0.4%).

### 2.12. Cutinase Activity Assay

Cutin was isolated from apple peels [36]. To induce cutinase production in the fungus, 1 mL of conidial suspension (1 × 10^6^ /mL) was added to 100 mL of the modified Czapek–Dox broth medium (cutin 1.00 g/L, NaNO_3_ 3.00 g/L, K_2_HPO_4_, MgSO_4_·7H_2_O 0.50 g/L, KCl 0.50 g/L, and FeSO_4_ 0.01 g/L) and incubated at 200 rpm at 28 °C for 7 days.

The cutinase activity was measured as described in [37], with slight modifications. The enzyme activity assay reaction system contained 200 μL of fungal culture medium after the removal of conidia and mycelium, 690 μL of 100 mM sodium phosphate buffer (pH 7.0), 100 μL of 0.8% Triton X-100, and 20 μL of 1.76% (*v*/*v*) ρ-nitrophenyl butyrate (PNB) in acetonitrile. The reaction was set at 37 °C for 10 min. The PNB was hydrolyzed into p-nitrophenol by cutinase enzymes. The absorbance at 405 nm was measured with a microplate reader (TECAN, Grödig, Austria). The molar extinction coefficient for PNB was 6.83 × 10^3^ (pH 7.0).

### 2.13. Statistical Analysis

The statistical graphs were created using GraphPad Prism 8.0.2. One-way analysis of variance and least significant difference analyses were performed using IBM SPSS Statistics 26, with *p* < 0.05, *p* < 0.01, and *p* > 0.05 defined as statistically significant, extremely significant, and no significant difference, respectively.

## 3. Results

### 3.1. Identification of Cutinase G-Box Binding Protein FsCGBP in F. sacchari

**Figure 1:**
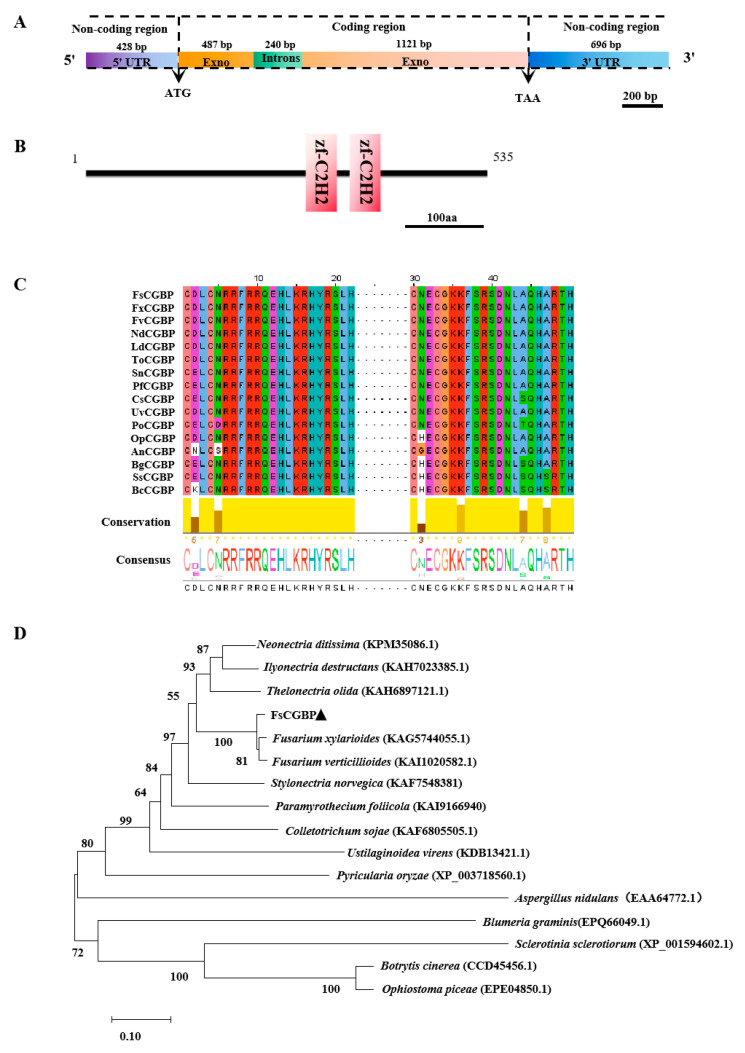
FsCGBP structure and its evolutional relationship to other fungal CGBP proteins. (**A**) Gene structure of FsCGBP. (**B**) FsCGBP protein contains two C2H2 conserved domains. (**C**) Alignment of conserved ZnF-C2H2 domains among CGBP homologs from fungal species. CGBP sequences were downloaded from the NCBI database. FxCGBP: *F. xylarioides* (KAG5744055.1), FvCGBP: *F.verticillioides* (KAI1020582.1), NdCGBP: *Neonectria ditissima* (KPM35086.1), LdCGBP: *Ilyonectria destructans* (KAH7023385.1), ToCGBP: *Thelonectria olida* (KAH6897121.1), SnCGBP: *Stylonectria norvegica* (KAF7548381), PfCGBP: *Paramyrothecium foliicola* (KAI9166940), CsCGBP: *Colletotrichum sojae* (KAF6805505.1), UvCGBP: *Ustilaginoidea virens* (KDB13421.1), PoCGBP: *Pyricularia oryzae* (XP_003718560.1), OpCGBP: *Ophiostoma piceae* (EPE04850.1), AnCGBP: *Aspergillus nidulans* (EAA64772.1), BgCGBP: *Blumeria graminis* (EPQ66049.1), SsCGBP: *Sclerotinia sclerotiorum* (XP_001594602.1), BcCGBP: *Botrytis cinerea* (CCD45456.1). (**D**) Phylogenetic tree of CGBP orthologs. The neighbor-joining tree was generated using Mega 7.0 with 1000 bootstrap replicates.

The virulence-defective mutant FsAT0345, which was screened from a random insertion mutant library, was mapped to the coding region in a putative cutinase G-box binding protein gene (GenBank accession number: OR752392) in our previous study [28] and annotated as *FsCGBP*. The alignment of the genomic DNA and cDNA sequences of the *FsCGBP* gene revealed that it contains a 1608 bp coding sequence with an intron of 240 bp, encoding a protein composed of 535 amino acids with an apparent molecular weight of 57.77 kDa (Figure 1A). Protein domain analysis revealed that FsCGBP contains two ZnF-C2H2 conserved domains, which are highly conserved among different C2H2 TFs (Figure 1B,C). A BLAST search within the NCBI protein database revealed FsCGBP homologous proteins to be widely distributed and highly conserved in ascomycetes. The search also showed that FsCGBP shares 54.0% identity in the amino acid sequence with UvCGBP, which was identified and characterized as a cutinase G-box binding protein in *Ustilaginoidea virens* in a recent study [27]. Phylogenetic analysis unveiled that FsCGBP is most similar to the CGBP protein of *F. xylarioides* and *F. verticillioides*, sharing 95.9% and 94.3% identities in amino acid sequences, respectively (Figure 1D). The transactivation activity was then examined, and the result showed that FsCGBP exhibited a strong transcriptional activation activity in yeast (Figure S1).

### 3.2. FsCGBP Is Localized in the Nucleus

**Figure 2:**
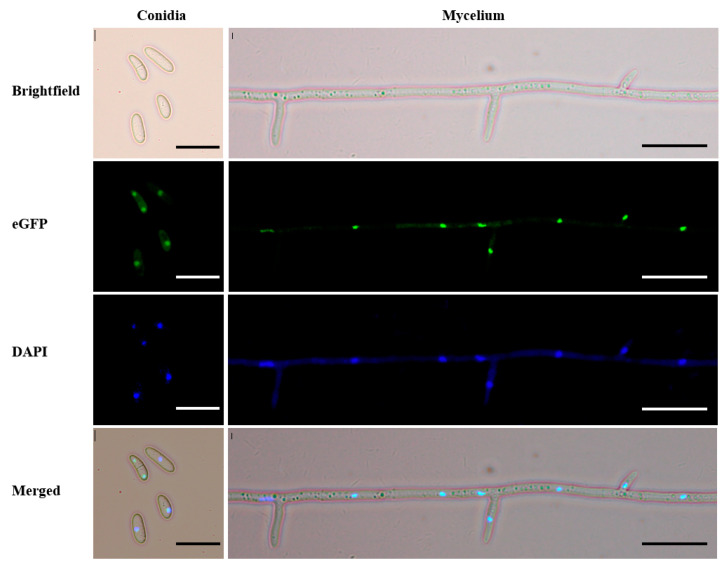
Subcellular localization of FsCGBP in *F. sacchari*. The nuclei were stained with DAPI and examined using fluorescence microscopy. Scale bar = 20 μm.

To determine the subcellular localization of FsCGBP in *F. sacchari*, an eGFP-FsCGBP fusion protein-expressing plasmid (pCPXHY2-eGFP-*FsCGBP*) was constructed and transformed into the *F. sacchari* WT strain FF001. The GFP fluorescence was found to localize in the nuclei of conidia and mycelium using fluorescence microscopy. The GFP signal colocalized with the staining signal of the nucleus-specific dye DAPI, confirming that FsCGBP is indeed localized in the *F. sacchari* nucleus (Figure 2).

### 3.3. FsCGBP Contributes to Mycelium Growth, Conidiogenesis, and Conidial Germination

**Figure 3:**
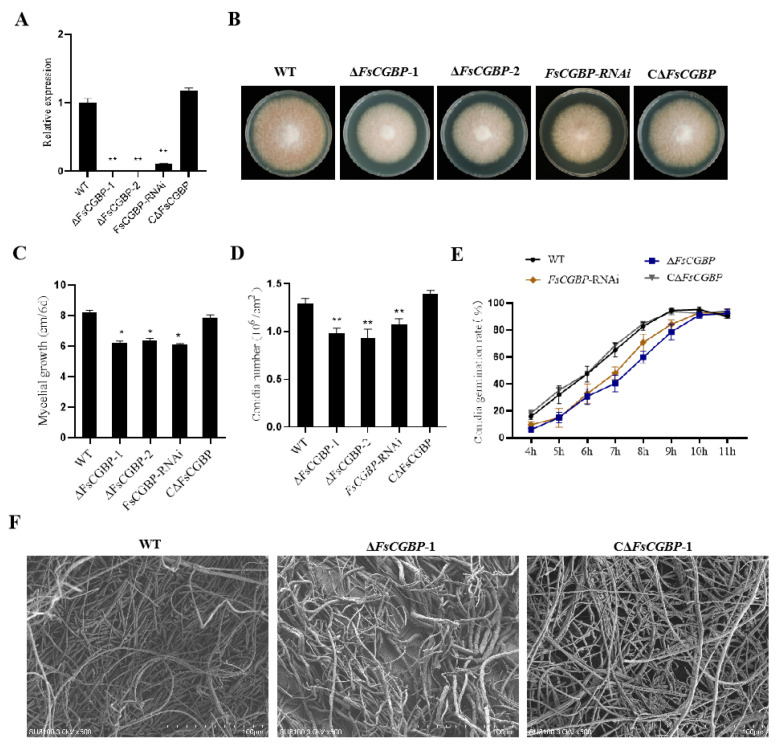
FsCGBP contributes to mycelium growth, conidiogenesis, and conidial germination in *F. sacchari.* (**A**) Expression levels of the FsCGBP gene in the WT, Δ*FsCGBP*-1, Δ*FsCGBP*-2, *FsCGBP*-RNAi, and CΔ*FsCGBP* strains using qRT-PCR. (**B**) Colony morphology. (**C**) Colony diameter. (**D**) Conidial production (10^6^/cm^2^) of the WT, Δ*FsCGBP*-1, Δ*FsCGBP*-2, *FsCGBP*-RNAi, and CΔ*FsCGBP* strains on PDA medium at 28 °C in the dark at 6 days post-inoculation (dpi). (**E**) Conidial germination rate on agar medium at 28 °C for 11 h. (**F**) SEM analysis of the mycelium of the WT, ∆*FsCGBP*, and C∆*FsCGBP*. * Represents a significant difference (*p* < 0.05), ** represents an extremely significant difference (*p* < 0.01).

To investigate the functions of FsCGBP in *F. sacchari*, the deletion mutants Δ*FsCGBP*-1, Δ*FsCGBP*-2, silencing strain *FsCGBP*-RNAi, and complementation strain CΔ*FsCGBP* were constructed and confirmed using PCR and RT-PCR (Figure S2). The expression of FsCGBP was not detected in Δ*FsCGBP*-1 and Δ*FsCGBP*-2, while a significantly reduced level (10.45% of the WT) was found in the *FsCGBP*-RNAi strain. As expected, its expression level was fully recovered in the complementation strain CΔ*FsCGBP* (Figure 3A).

∆*FsCGBP-*1, ∆*FsCGBP-*2, and *FsCGBP*-RNAi exhibited a significant reduction in the mycelial growth rate compared with those of the WT and C∆*FsCGBP*, with the colony diameter decreasing by approximately 19.31% (Figure 3B,C). Compared with those of the WT and C∆*FsCGBP*, the conidiogenesis of ∆*FsCGBP* was drastically inhibited, with conidial production decreasing by approximately 77.62% (Figure 3D). The conidial germination of Δ*FsCGBP* and *FsCGBP*-RNAi was delayed in agar media compared with those of the WT strain FF001 and C∆*FsCGBP*. In particular, the conidial germination of the WT strain FF001 and C∆FsCGBP almost peaked at 8 hpi, with a germination rate of more than 92.00%, while those of Δ*FsCGBP* and *FsCGBP*-RNAi were 78.33% and 66.80%, respectively (Figure 3E). Moreover, SEM revealed that the mycelium of ∆*FsCGBP* was thin, tangled, and with abnormal growth. In contrast, the mycelium of the WT was smooth, long, and straight with uniform thickness (Figure 3F).

### 3.4. FsCGBP Is Involved in Resistance to Abiotic Stresses

**Figure 4:**
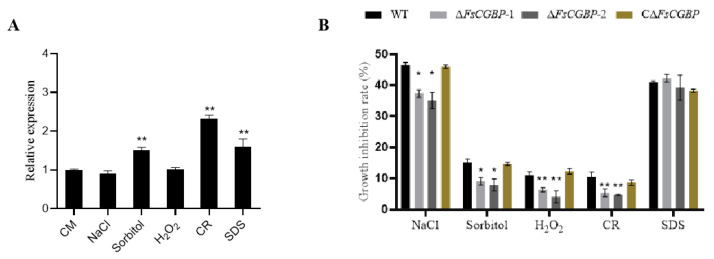
*FsCGBP*-deletion mutant showed increased tolerance to oxidative, osmotic, and cell wall stresses. (**A**) Colony morphology of the WT strain FF001 and mutant strains on CM supplemented with 1 M NaCl, 1 M sorbitol, 0.05% H_2_O_2_ (*v*/*v*), 0.3 mM CR, and 0.5 mM SDS at 28 °C in the dark at 4 days post-inoculation (dpi). (**B**) Relative area rates of the WT strain FF001 and mutant strains at 28 °C in the dark at 4 dpi. * Represents a significant difference (*p* < 0.05) and ** represents an extremely significant difference (*p* < 0.01).

The transcription of *FsCGBP* was upregulated in response to stress by sorbitol, CR, and SDS but not NaCl and H_2_O_2_ (Figure 4A). To explore the role of FsCGBP in stress response, an ∆*FsCGBP* mutant was inoculated in a CM medium complemented with various stress elements. The mutant grew significantly more slowly in the presence of NaCl, sorbitol, H_2_O_2_, and CR than the WT and complemented strains (Figure 4B).

### 3.5. Deletion of FsCGBP Attenuates the Virulence of F. sacchari

**Figure 5:**
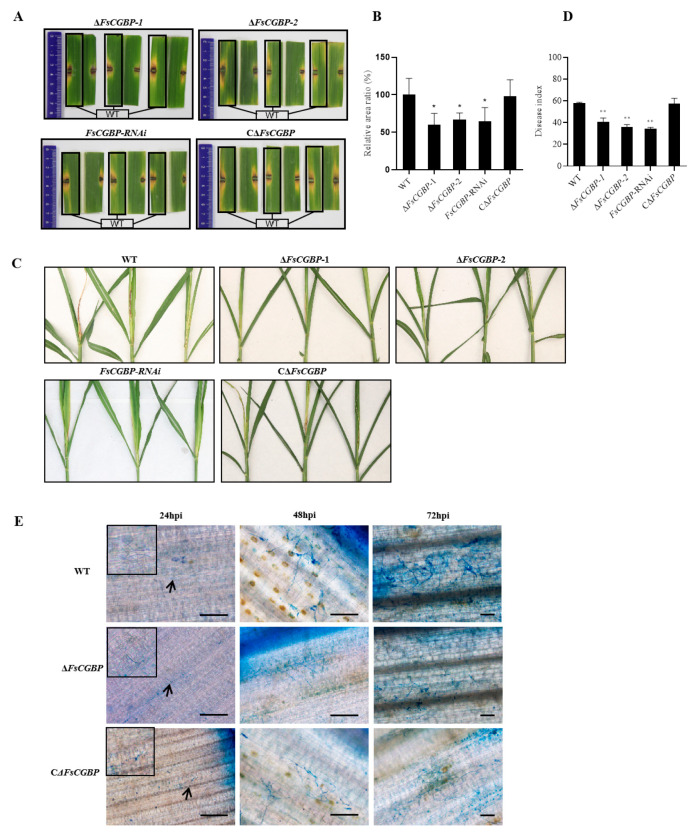
Deletion of FsCGBP attenuates the virulence of *F. sacchari*. (**A**) Disease symptoms, (**B**) relative lesion area ratio on the detached leaves inoculated with the mycelial plug of the WT strain FF001 (boxed), ∆*FsCGBP*-1, ∆*FsCGBP*-2, ∆*FsCGBP*-RNAi, and C∆*FsCGBP* at 4 dpi. (**C**) Disease symptoms on the sugarcane plants inoculated by injection at 14 dpi. (**D**) DSI of sugarcane plants at 14 dpi. (**E**) Microscopy of fungal mycelia in the inoculated sugarcane leaves. The leaf tissue sections were stained with trypan blue. Scale bar = 50 μm. * Represents a significant difference (*p* < 0.05), ** represents an extremely significant difference (*p* < 0.01).

The expression level of *FsCGBP* was lower than that of the conidia throughout the germination process of *F. sacchari,* while its expression increased significantly at 24–96 hpi (Figure S3). To investigate the role of FsCGBP in *F. sacchari* virulence, virulence assays were first performed with the detached leaves in vitro. The WT strain FF001 and C∆FsCGBP caused typical yellow lesions, while the lesions caused by the Δ*FsCGBP* and *FsCGBP*-RNAi were significantly smaller than those of the WT strain FF001 and C∆FsCGBP (Figure 5A,B). The attenuated virulence of the FsCGBP mutant was further validated on whole sugarcane plants. At 14 dpi, the sugarcanes inoculated with WT and CΔ*FsCGBP* showed severe necrosis, distortion, and yellowing at the plant tops. However, while sugarcane plants inoculated with the Δ*FsCGBP* strain also exhibited slight twisting and yellowing, necrosis was minimal (Figure 5C,D). Histopathological examination revealed that Δ*FsCGBP* infiltrated much slower than the WT or CΔ*FsCGBP* and generated a limited number of invasive hyphae inside the sugarcane leaves, contrasting with the WT and CΔ*FsCGBP*, which produced a large number of infectious hyphae at 48 hpi (Figure 5E). This result suggests the decreased virulence in Δ*FsCGBP* can be attributed to delayed germination and slow mycelial growth.

### 3.6. FsCGBP Regulates the Transcription of Cutinase Genes

**Figure 6:**
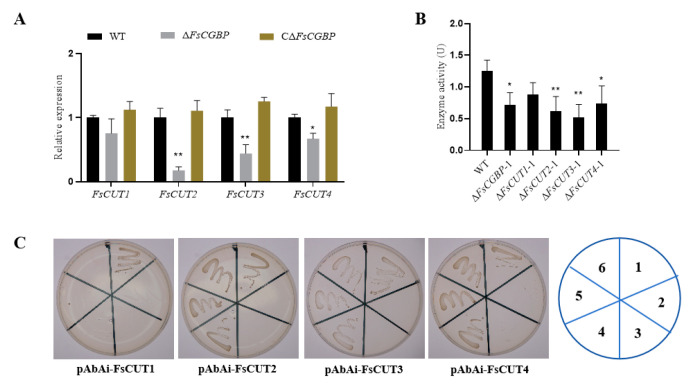
FsCGBP transcriptionally regulates the expression of cutinase genes in *F. sacchari*. (**A**) Expression of four cutinase genes in the WT strain FF001, ∆*FsCGBP*, and C∆*FsCGBP* using qRT-PCR. (**B**) Cutinase activity of WT, Δ*FsCGBP*, Δ*FsCUT1,* Δ*FsCUT2,* Δ*FsCUT3,* and Δ*FsCUT4* in modified Czapek–Dox broth for 7 days. (**C**) Interactions of FsCGBP and the promoter of FsCut2, FsCut3, or FsCut4 were analyzed by yeast one-hybrid assays. The transformed yeast containing the indicated plasmids was streaked on an SD/-Leu-Ure selective medium (supplemented with 500 ng/mL AbA), respectively. 1: p53-AbAi+pGADT7-p53 (positive control). 2: p53-AbAi + pGADT7-FsCGBP (negative control). 3: pAbAi-pro+ pGADT7 (negative control). 4~6: pAbAi-pro+pGADT7-FsCGBP. * Represents a significant difference (*p* < 0.05), ** represents an extremely significant difference (*p* < 0.01).

It has been reported that CGBP is a cutinase G-box binding protein [27]. In *U. virens*, cutinase gene transcription levels were downregulated in ∆UvCGBP1-33 [27]. We identified four cutinase genes in the genome of the *F. sacchari* WT strain FF001, designated as *FsCUT1*~*FsCUT4* (GenBank accession numbers: OR752396, OR752397, OR752398, and OR752396). Quantification of mRNA using qRT-PCR revealed that the expression levels of *FsCUT2*, *FsCUT3*, and *FsCUT4* were significantly downregulated in Δ*FsCGBP*, while *FsCUT1* had a similar expression level to that of the WT strain FF001 (Figure 6A). To confirm the contribution of each of the cutinase genes that FsCGBP regulated to the cutinase activity in the fungus, we constructed *FsCUT* deletion mutants (Figure S2F) and measured their total cutinase enzyme activity. With the WT strain FF001as reference, deletion of *FsCGBP*, *FsCUT2*, *FsCUT3*, and *FsCUT4* reduced the cutinase activity by 42.4%, 50.4%, 58.6%, and 41.2%, respectively (Figure 6B), implying that the reduced cutinase activity in ΔFsCGBP was linked to the downregulated expression of these *FsCUTs*.

The G-box elements in the promoter region in the *FsCUT*s were predicted using the online PlantCARE tool (http://bioinformatics.psb.ugent.be/webtools/plantcare/html/) (Figure S4), and yeast one-hybrid assays determined their interaction with FsCGBP. Except for *FsCUT 1*, constructs containing *FsCGBP* and the G-box fragment in the promoter region in *FsCUT2*, *FsCUT3*, or *FsCUT4* grew well on SD/-Leu-Ure selective medium (Figure 6C). These results indicate that three out of four cutinase genes could be transcriptionally activated by FsCGBP, likely by binding to the G-box elements. However, no significant differences were observed between the *FsCUTs* deletion mutants Δ*FsCUT2*, Δ*FsCUT3,* or Δ*FsCUT4* and the WT strain FF001 in terms of conidial production, conidial germination, virulence, and sensitivity to various abiotic stresses (Figure S5B,C), indicating that alternation of phenotypes in Δ*FsCGBP* is independent of a reduction in cutinase.

### 3.7. FsCGBP Regulates the Transcription of Key Protein Kinase Genes in the MAPK Pathway

**Figure 7:**
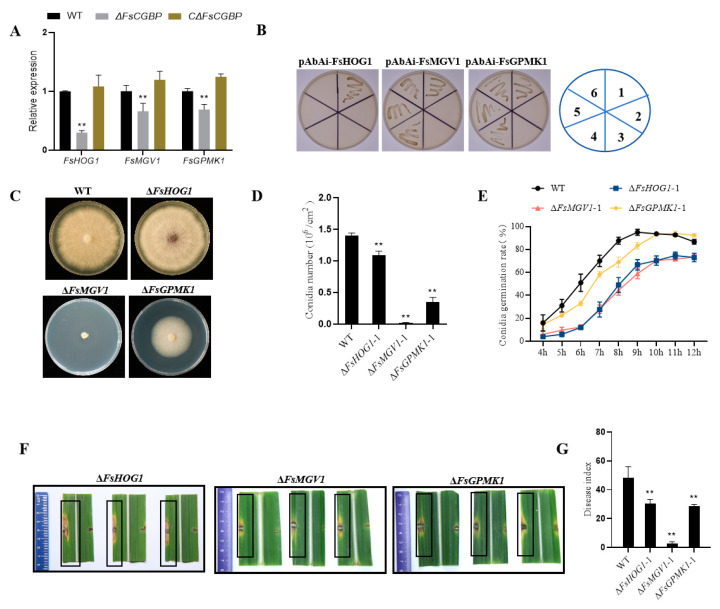
FsCGBP functions in the development and pathogenicity of *F. sacchari* by regulating the MAPK pathway. (**A**) Expression of *FsHOG1*, *FsMGV1,* and *FsGPMK1* genes in the WT strain FF001, Δ*FsCGBP* strain, and CΔ*FsCGBP* strain using qRT-PCR. (**B**) Interactions of FsCGBP and the promoter of *FsHOG1*, *FsMGV1*, or *FsGPMK1* were analyzed using yeast one-hybrid assays. The transformed yeast containing the indicated plasmids was streaked on an SD/-Leu-Ure selective medium (supplemented with 500 ng/mL AbA), respectively. 1: p53-AbAi+pGADT7-p53 (positive control). 2: p53-AbAi + pGADT7-FsCGBP (negative control). 3: pAbAi-pro+ pGADT7 (negative control). 4~6: pAbAi-pro + pGADT7-FsCGBP. (**C**) Colony morphology and (**D**) colony diameter of the WT, Δ*FsHOG1,* Δ*FsMGV1,* and Δ*FsGPMK1* strains on PDA medium at 28 °C in the dark at 6 dpi. (**E**) Conidia germination rate of the WT, Δ*FsHOG1,* Δ*FsMGV1*, and Δ*FsGPMK1* on agar medium at 28 °C for 10 h. (**F**) Disease symptoms on the detached leaves inoculated with the mycelial plug of the WT strain FF001 (in boxes), Δ*FsHOG1,* Δ*FsMGV1*, and Δ*FsGPMK1* strains at 4 dpi. (**G**) DSI of sugarcane plants of the WT strain FF001, Δ*FsHOG1,* Δ*FsMGV1*, and Δ*FsGPMK1* strains by the syringe inoculation method at 14 dpi. ** represents an extremely significant difference (*p* < 0.01).

The MAPK pathway was reported to be important for the virulence of various pathogenic plant fungi [38]. Chen et al. revealed Uv*CGBP*1 functions to regulate the virulence *of U. virens* via MAPK pathways, and the expression of UvPmk1 and UvSlt2 was significantly reduced in the ∆UvCGBP1 mutant during infection at 3 dpi [27]. To explore the functional link between FsCGBP protein and the MAPK pathway in *F. sacchari*, we conducted qRT-PCR and yeast one-hybrid assays and found that the expressions of *FsHOG1* (GenBank accession number: OR752393), *FsMGV1* (GenBank accession number: OR752394), and *FsGPMK1* (GenBank accession number: OR752395) encoding the key protein kinases in the MAPK pathway were all downregulated significantly in the ∆*FsCGBP* (Figure 7A). Yeast one-hybrid assays confirmed that FsCGBP was able to activate the transcription of *FsMGV1* and *FsGPMK1* but not *FsHOG1* (Figure 7B).

To further investigate the roles of the *FsHOG1*, FsMGV1, and FsGPMK1 in *F. sacchari*, we constructed their mutant strains (Figure S2G) and performed phenotypic and virulence analyses. The deletion of *FsGPMK1* resulted in extremely severe inhibition of mycelial growth, conidial production, and germination, and almost a total loss of virulence; deletion of *FsMGV1* or *FsHOG1* significantly compromised the above phenotypes, but to a lesser extent (Figure 7C–G). These results demonstrate that *FsMGV1* is the major contributor to the regulation of the essential phenotypes and virulence, suggesting that *FsCGBP* may very likely exert its impact through its regulation of *FsMGV1*.

## 4. Discussion

Although *F. sacchari* has long been identified as the predominant causal agent for PBD, knowledge of its virulence regulation is limited. In this study, we identified a homolog of the C2H2-type TF in *F. sacchari*, FsCGBP, and confirmed that it was capable of activating the transcription of targeted genes in vitro (Figure S1). Similarly to other C2H2 TFs, such as UvCGBP1 in *U. virens,* VdCf2 in *V. dahliae*, PlCZF1 in *Peronophythora litchii*, and CfCpmd1 in *Colletotrichum fructicola* [24,25,26,27], FsCGBP was shown to play essential roles in mycelial growth, conidiogenesis, conidial germination, stress resistance, and virulence in *F. sacchari* (Figure 3, Figure 4, Figure 5 and Figure 6).

In *U. virens,* the expression of UvCGBP1 was upregulated under NaCl osmotic stress and H_2_O_2_ oxidative stress. Moreover, ∆*UvCGBP1* mutants were more sensitive to NaCl, sorbitol, and SDS [27]. Although the expression level of FsCGBP did not change under NaCl and H_2_O_2_ stress (Figure 4A), the deletion of FsCGBP resulted in a significant reduction in the growth rate of the mutant in media supplemented with NaCl, sorbitol, H_2_O_2_, or CR in *F. sacchari* (Figure 4B), indicating that FsCGBP and UvCGBP1 exhibit significantly different responses to abiotic stress, implying different regulatory networks between the two fungi.

MAPK cascade pathways, which mediate cellular responses to environmental and physiological signals in eukaryotes, are broadly conserved [22]. Three types of MAPK signaling pathways have been reported in plant pathogenic fungi, namely, the FUS3/KSS1, SLT2, and HOG1 pathways, which play crucial roles in fungal development, stress responses, and virulence [22,38,39,40,41,42,43]. In this study, the transcription levels of *FsHOG1*, *FsMGV1*, and *FsGPMK1* in the ∆*FsCGBP* strains were significantly lower than those in the WT strain FF001. Furthermore, *FsMGV1* and *FsGPMK1* separately interacted with FsCGBP via its promotor in the yeast cells. Noticeably, the deletion of *FsGPMK1* or *FsMGV1* caused obvious defects in fungal growth, conidiation, and virulence compared with that of the WT (Figure 7C–G). Therefore, we propose that the *FsCGBP* exerts its functions by regulating the transcription of *FsMGV1* and *FsGPMK1*. The HOG pathway is not only related to environmental stress, but also regulates the pathogenicity in plant pathogenic fungi [44,45,46,47]. In this study, the expression of *FsHOG1* was significantly downregulated in Δ*FsCGBP*, but FsCGBP did not directly interact with the FsHOG1. We speculate that FsCGBP may be involved in the regulation of FsHog1 upstream genes, such as Pbs2, Ssk22 [48], and Ste11 [49], and ultimately affect the expression of *FsHog1*. Further research is needed to verify the function of the HOG1 pathway in the pathogenicity and the interaction between its upstream genes and *FsCGBP*.

The plant cuticle, which constitutes the first physical defensive barrier against environmental stresses and pathogen invasion, is mainly composed of cutin [42,50]. As a member of the a/β hydrolase superfamily, cutinases can hydrolyze cutin, which facilitates fungal penetration into their hosts, and play a critical role in plant surface signaling [42,51]. Cutinases have been shown to be a virulence factor in some plant pathogens [50,52]. In contrast, for other pathogenic fungi, the cutinase is dispensable due to its pathogenicity [27,53]. In this study, the deletion of individual cutinase genes resulted in a reduction in the cutinase activity in *F. sacchar*i, but this was not the case for virulence. In addition to the cutinase genes investigated in this study, six “cutinase-relate genes” were found in the genome of *F. sacchari* (our unpublished data). We speculate that the functions of the cutinase genes or cutinase-related genes are redundant. In future research, it would be desirable to spontaneously knock multiple cutinase genes in *F. sacchari* to verify their involvement during the infection process. FsCGBP can bind the promoter of at least three cutinase genes, and the cutinase activity of ΔFsCGBP was 57.62% that of the WT strain, which is higher than that of the cutinase gene deletion mutants. Hence, the decline in ΔFsCGBP virulence is independent of the reduction in the cutinase activity. The ΔFsCGBP mutant showed a significant reduction in mycelial growth, and a delay in conidial germination, indicating that its attenuated virulence is due to impaired development.

Host-induced gene silencing is an effective strategy for the improvement of plant resistance to pathogens by silencing genes essential for the pathogenicity of the pathogens, and it has been shown to be successful in a number of plant–pathogen systems [54,55,56]. As a prerequisite, the target gene selected for RNAi must be a key pathogenicity/virulence gene and will have no, or minimal, side effects on off-target organisms [55]. In this regard, *FsCGBP* or *FsMGV1* seems to meet this criterion of RNAi targets. Moreover, silencing of FsCGBP resulted in a reduction in mycelial growth and virulence in the *F. sacchar*. As FsCGBP is ascomycete-specific and has not been found in plants, it may have the potential to create PBD-resistant transgenic sugarcanes.

## 5. Conclusions

In this study, we functionally characterized FsCGBP, a cutinase G-box binding protein in *F. sacchari*. Our results indicated FsCGBP to be a C2H2-type zinc finger protein that is important for vegetative growth, conidia production, conidial germination, and responses to the different stresses and *F. sacchari* virulence. FsCGBP was observed to interact with three cutinase genes (*FsCUT2*, *FsCUT3*, and *FsCUT4*) and regulated their expression levels. More importantly, FsCGBP could also directly interact with the MAPK-related genes (*FsMGV1* and *FsGPMK1*) and function using the MAPK pathway to regulate the development and *F. sacchari* virulence. This study provides new insights into the mechanisms underlying the development and virulence regulation in *F. sacchari*.

## Data Availability

Data are contained within the article and Supplementary Materials.

## Supplementary Materials

The following supporting information can be downloaded at: https://www.mdpi.com/article/10.3390/jof10040246/s1. Figure S1: Transcriptional activity of FsCGBP in yeast cells; Figure S2: Generation and complementation of the mutants; Figure S3: Expression levels of *FsCGBP*; Figure S4 Construction of the pAbAi-pro bait vectors; Figure S5: Phenotype and virulence of the FsCUTs deleted mutants. Table S1: Fungal strains used in this study; Table S2: Plasmids used in this study; Table S3: Primers used in this study.
