# Supplementary material for: FsCGBP, a Cutinase G-Box Binding Protein, Regulates the Growth, Development, and Virulence of Fusarium sacchari, the Pathogen of Sugarcane Pokkah Boeng Disease
Source: J Fungi (Basel). 2024 Mar 25;10(4):246. doi: 10.3390/jof10040246 (PMC11051240; doi:10.3390/jof10040246)
Supplement: Supplementary file 1 [file jof-10-00246-s001.zip › jof-2881116-supplementary.pdf]

Supplementary Materials (Liang et al. FsCGBP, a cutinase G-box binding protein, regulates the growth, development, and virulence of *Fusarium sacchari*, the pathogen of sugarcane Pokkah boeng disease)

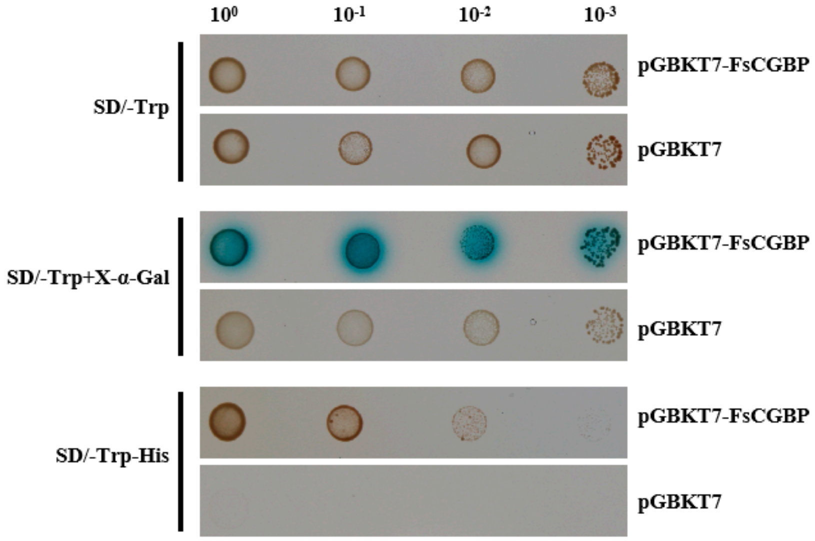

Figure S1. Transcriptional activity of FsCGBP in yeast cells.

Yeast cells were assayed on SD-Trp, SD-Trp-His, and SD-Trp /X-a-Gal medium. The yeast suspension aliquots were diluted to 10^{-1}, 10^{-2}, and 10^{-3}, and 10 μl of each dilutant was grown on the plates. The yeast transformants containing the pGBKT plasmid were used as a negative control.

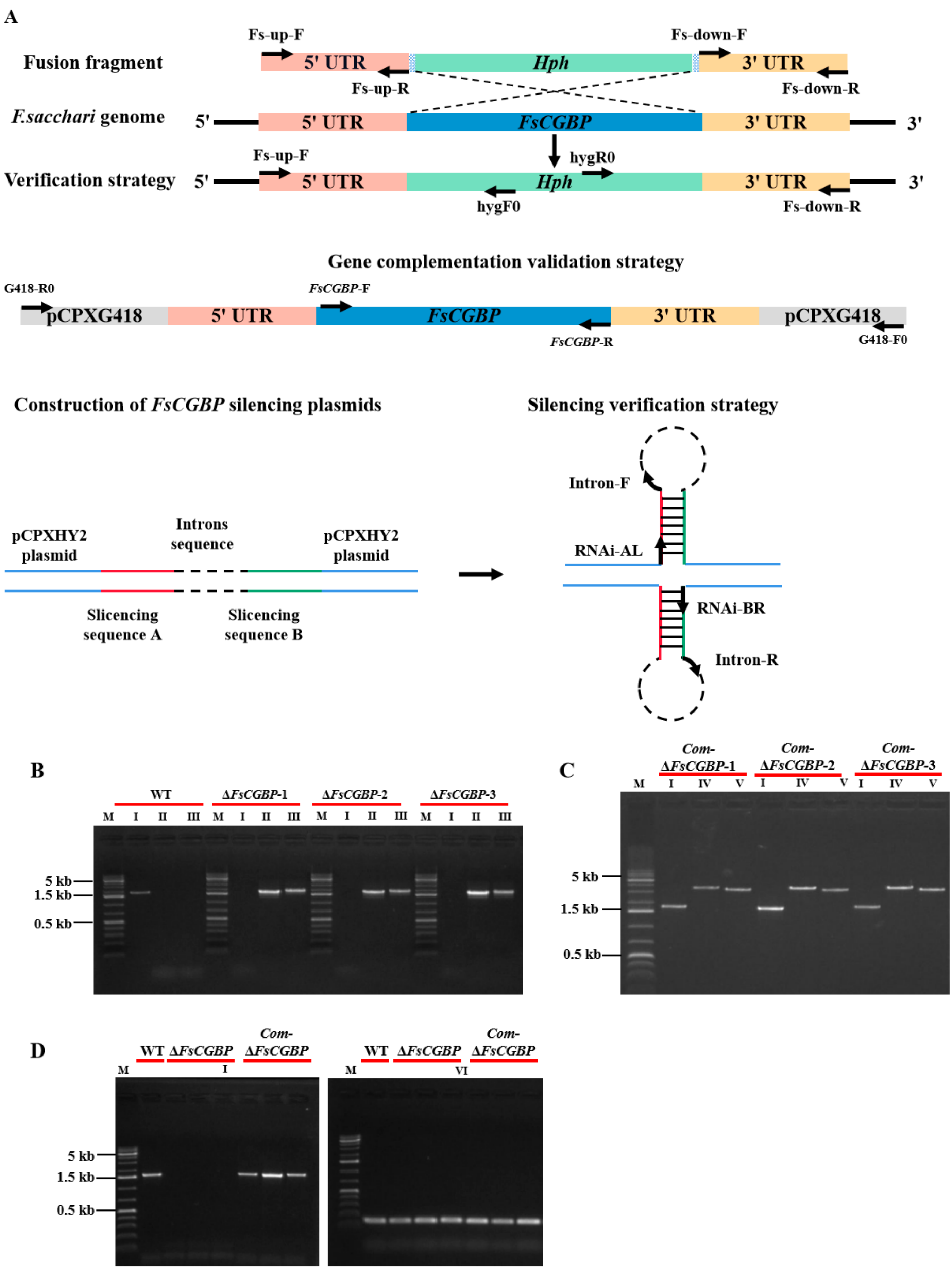

A
Fusion fragment
Fs-up-F
5' UTR
Hph
3' UTR
Fs-down-F
Fs-up-R
Fs-down-R
F.sacchari genome
5'
5' UTR
FsCGBP
3' UTR
3'
Verification strategy
Fs-up-F
hygR0
hygF0
Fs-down-R
Gene complementation validation strategy
G418-R0
FsCGBP-F
pCPXG418
5' UTR
FsCGBP
3' UTR
pCPXG418
FsCGBP-R
G418-F0
Construction of FsCGBP silencing plasmids
Silencing verification strategy
pCPXHY2 plasmid
Introns sequence
pCPXHY2 plasmid
Slicencing sequence A
Slicencing sequence B
Intron-F
RNAi-AL
RNAi-BR
Intron-R
B
WT
ΔFsCGBP-1
ΔFsCGBP-2
ΔFsCGBP-3
M I II III
5 kb
1.5 kb
0.5 kb
C
Com-ΔFsCGBP-1
Com-ΔFsCGBP-2
Com-ΔFsCGBP-3
M I IV V
5 kb
1.5 kb
0.5 kb
D
WT
ΔFsCGBP
Com-ΔFsCGBP
M
I
VI
5 kb
1.5 kb
0.5 kb

(Figure S2 continued)

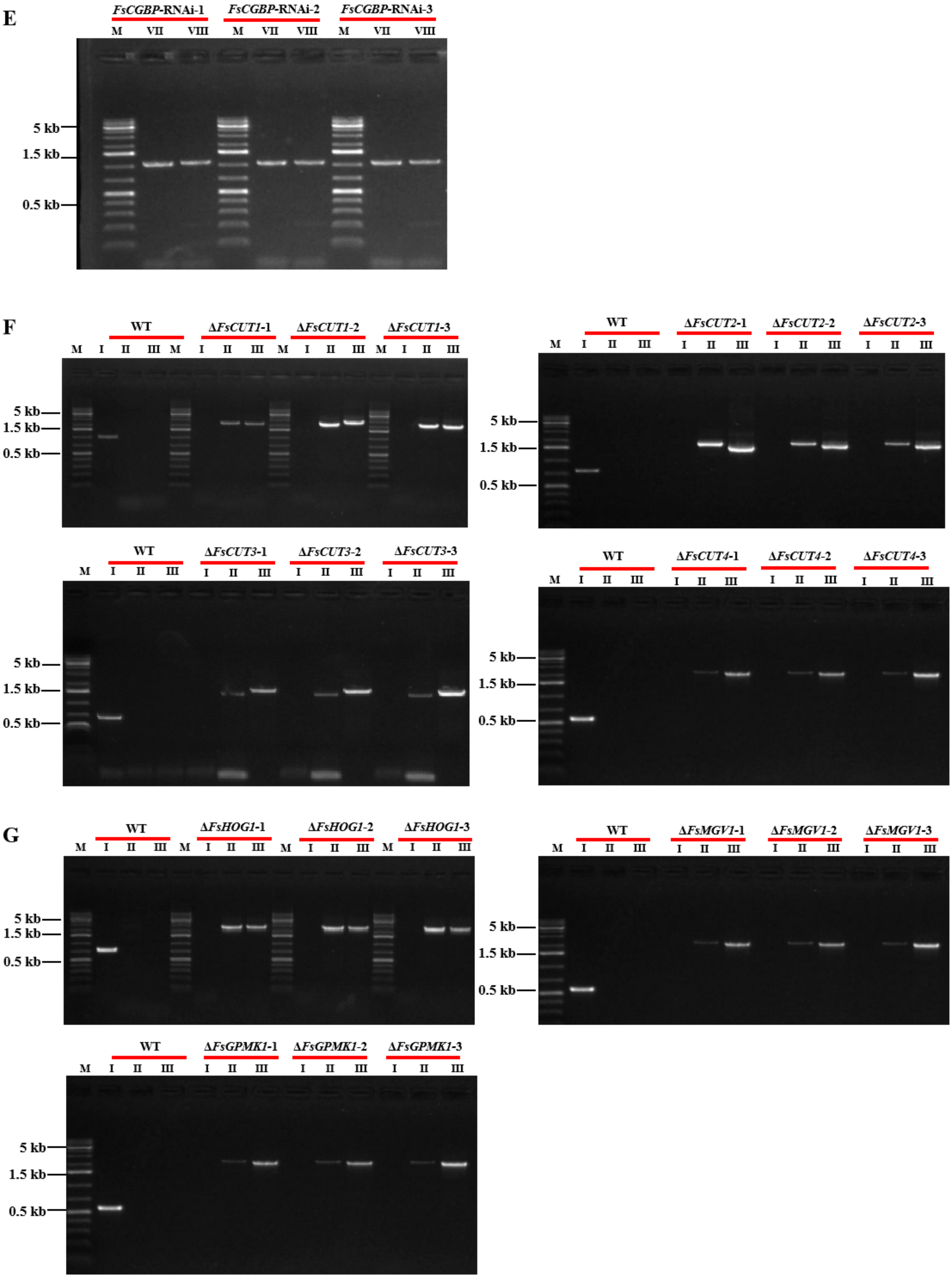

Figure S2. Generation and complementation of the mutants

Ⅰ: Fs-F/Fs-R. Ⅱ: Fs-up-F/hygF0. Ⅲ: Fs-down-R/hygR0. Ⅳ: *FsCGBP*-F/G418F0. Ⅴ: *FsCGBP*-R/G418R0. Ⅵ: qPCR-*Actin*-F/qPCR-*Actin*-R. Ⅶ: RNAi-AL/Intron-R. Ⅷ: RNAi-BR/Intron-F.

(A) Schematics of the construct strategy of the deletion mutant, complementation strain, and silencing strain. (B) Confirmed of *F. sacchari* FsCGBP deletions by genomic PCR using Fs-F/Fs-R, Fs-up-F/hygF0, and Fs-down-R/hygR0 primer pairs, respectively. (C) Confirmed of the complementation strains by PCR using Fs-F/Fs-R, *FsCGBP*-F/G418F0, and *FsCGBP*-R/G418R0 primer pairs, respectively. (D) Transcript levels of *FsCGBP* in WT strain FF001, *FsCGBP* deletions, and the complementation strains by RT-PCR using Fs-F/Fs-R, and the β-*Actin* gene was used as a control. (E) Confirmed silencing strains *FsCGBP*-RNAi by PCR using RNAi-AL/Intron-R and RNAi-BR/Intron-F primers, respectively. (F), (G) Confirmed of Δ*FsCut2,* Δ*FsCut3,* Δ*FsCut4,* Δ*FsHOG1,* Δ*FsMGV1,* and Δ*FsGPMK1* by PCR using Fs-F/Fs-R, Fs-up-F/hygF0, and Fs-down-R/hygR0 primer pairs, respectively. Fs-F/Fs-R is a general term for target gene detection primers, with their positions shown in Figure S2A and corresponding specific names shown in Table S2.

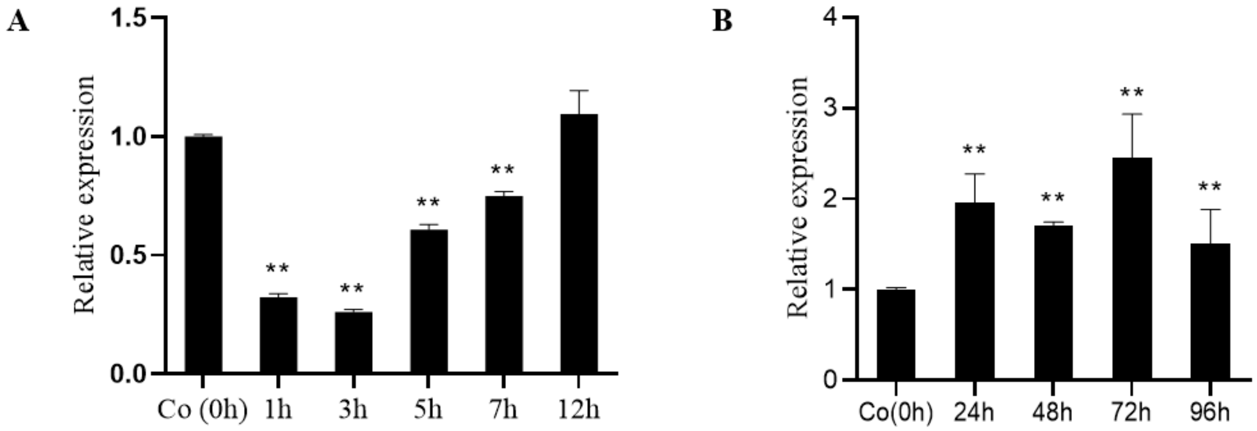

Figure S3. Expression levels of *FsCGBP*

(A) Expression levels of *FsCGBP* during conidia germination process in sugarcane leaf media. 1h, 3h, 5h, 7h and 12h represent the germination stages of *F. sacchari* at 1h, 3h, 5h, 7h, and 12h, respectively. (B) Expression levels of *FsCGBP* during the infection process. 24h, 48h, 72h, and 96h represent the infection stages of *F. sacchari* at 24, 48, 72, and 96 hpi, respectively. *β-Actin* gene was employed as the reference gene. Relative expression levels were calculated using the Co values as a reference. * represents a significant difference (P<0.05) and ** represents an extremely significant difference (P<0.01).

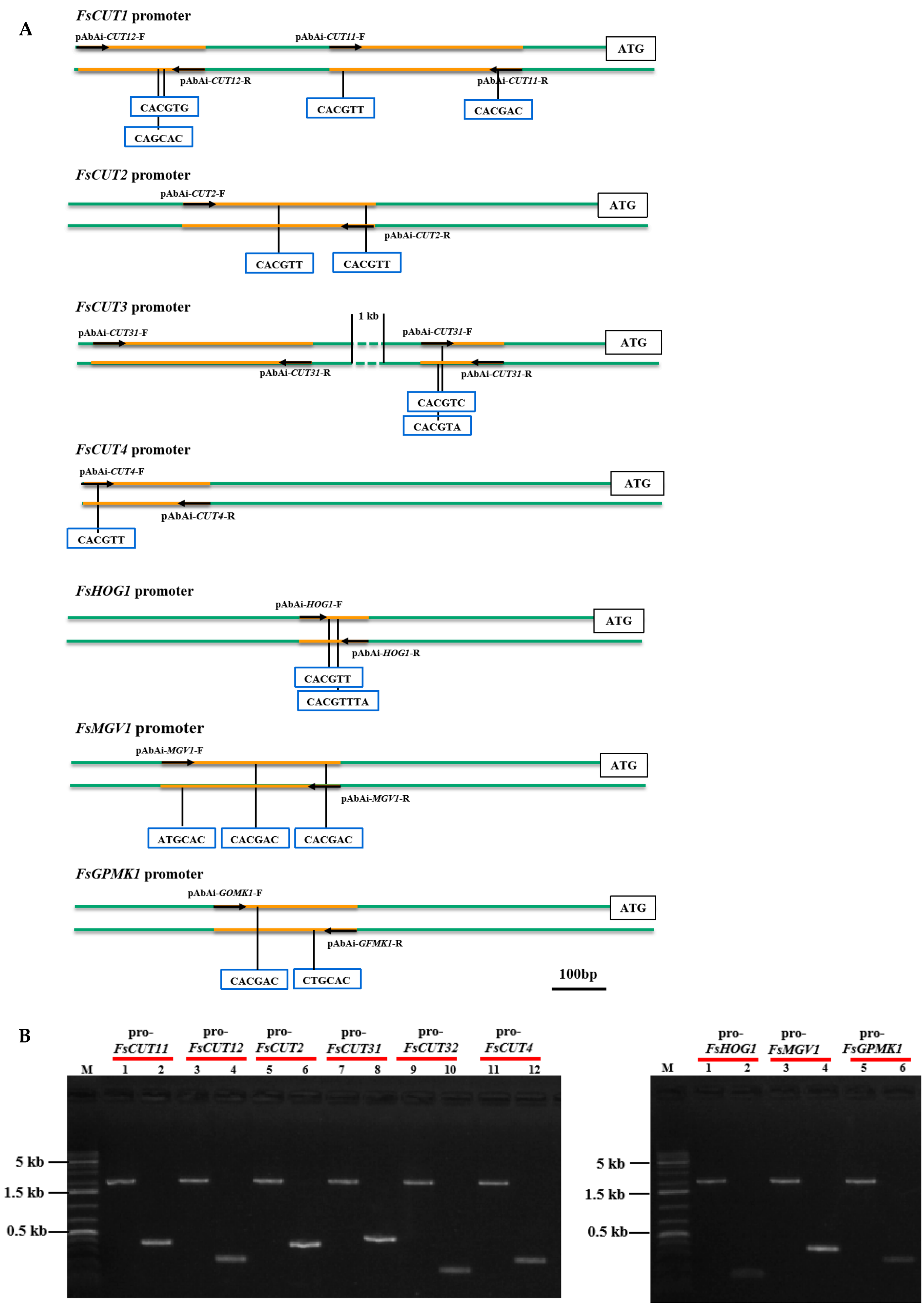
A
FsCUT1 promoter
pAbAi-CUT12-F
pAbAi-CUT11-F
ATG
pAbAi-CUT12-R
pAbAi-CUT11-R
CACGTG
CACGTT
CACGAC
CAGCAC
FsCUT2 promoter
pAbAi-CUT2-F
ATG
pAbAi-CUT2-R
CACGTT
CACGTT
FsCUT3 promoter
1 kb
pAbAi-CUT31-F
pAbAi-CUT31-F
ATG
pAbAi-CUT31-R
pAbAi-CUT31-R
CACGTC
CACGTA
FsCUT4 promoter
pAbAi-CUT4-F
ATG
pAbAi-CUT4-R
CACGTT
FsHOG1 promoter
pAbAi-HOG1-F
ATG
pAbAi-HOG1-R
CACGTT
CACGTTTA
FsMGV1 promoter
pAbAi-MGV1-F
ATG
pAbAi-MGV1-R
ATGCAC
CACGAC
CACGAC
FsGPMK1 promoter
pAbAi-GOMK1-F
ATG
pAbAi-GFMK1-R
CACGAC
CTGCAC
100bp
B
pro-FsCUT11
pro-FsCUT12
pro-FsCUT2
pro-FsCUT31
pro-FsCUT32
pro-FsCUT4
M 1 2 3 4 5 6 7 8 9 10 11 12
5 kb
1.5 kb
0.5 kb
pro-FsHOG1
pro-FsMGV1
pro-FsGPMK1
M 1 2 3 4 5 6
5 kb
1.5 kb
0.5 kb

Figure S4 Construction of the pAbAi-pro bait vectors (A) Schematics of the predicted FsCGBP binding promoters. The G-box element sequence in the promoters of *FsCUT2*, *FsCUT3*, *FsCUT4*, *FsHOG1, FsMGV1*, and *FsGPMK1,* is shown in the square frame respectively. Arrows display specific primers for amplifying the promoter region, with names consistent with Table S3. (B) PCR verification of yeast one-hybrid strains. M: GeneRuler™ 1kb Plus DNA ladder. 1, 3, 5, 7, 9, 11: *FsCGBP* target gene validation with *FsCGBP*-F/R. Other specific primer names and locations are shown in Figure S4A.

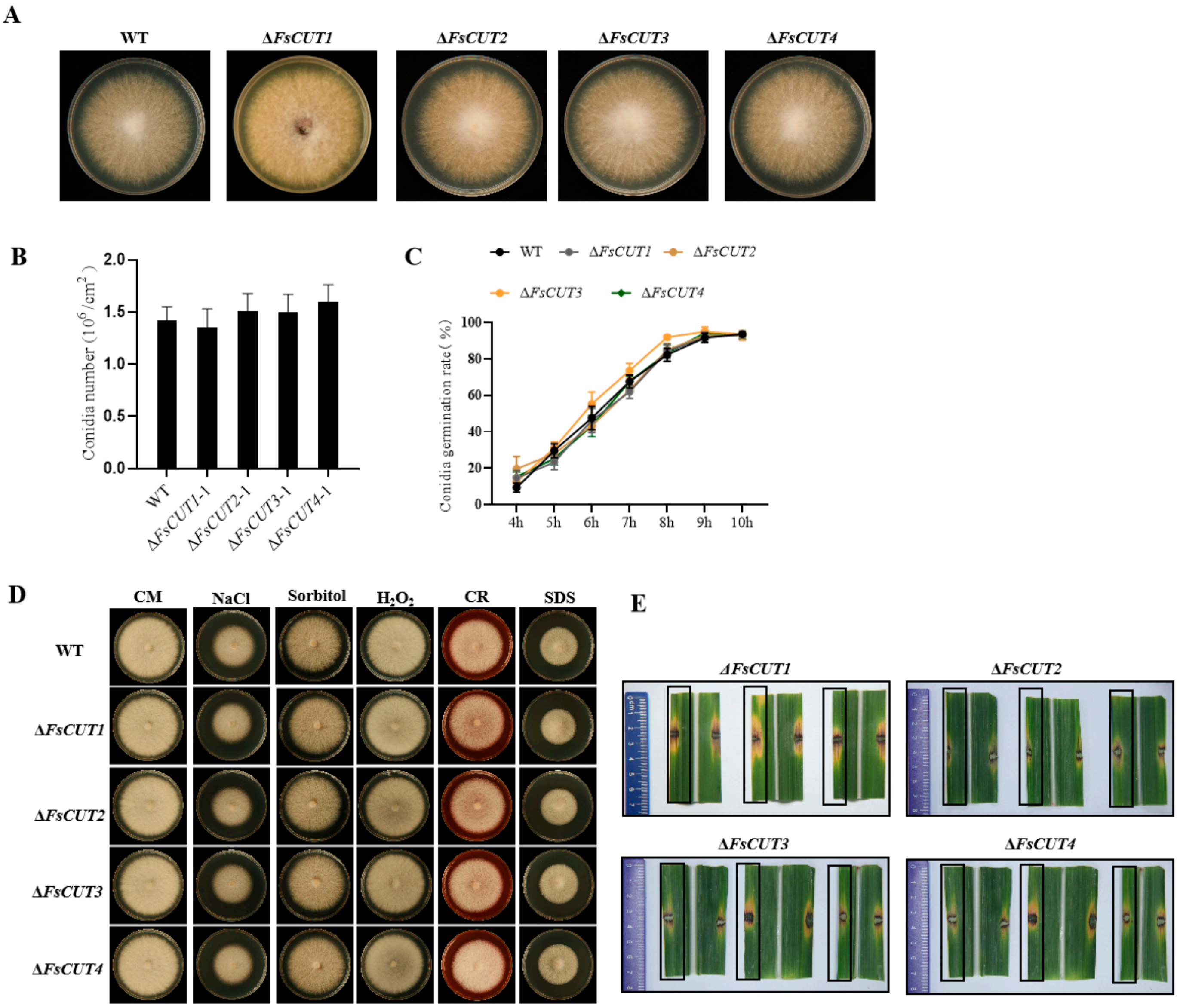

Figure S5. Phenotype and virulence of the *FsCut* deleted mutants. (A) Colony morphology. (B) Conidia number (10^6 /cm^2) of the WT, Δ*FsCUT2*, Δ*FsCUT3*, Δ*FsCUT4* strains on PDA medium at 28℃ for 6 days. (C) Germination rate of conidia on water agar medium at 28℃for 10 h. (D) Colony growth of the mutant strains on CM supplied with 1 M NaCl, 1 M Sorbitol, 0.05% H_2O_2 (v/v), 0.3 mM CR, 0.5 mM SDS after incubation at 28℃ for 4 days. (E) Disease symptoms on the detached leaves inoculated with the mycelial plug of the WT strain FF001(in box), Δ*FsCUT1*, Δ*FsCUT2*, Δ*FsCUT3*, Δ*FsCUT4* at 4 dpi.

Table S1 Fungal strains used in this study

Strain	Description	Source
WT (FF001)	Highly pathogenic *F. sacchari* strains	**[11]**
Δ*FsCGBP*-1	*FsCGBP* gene deletion mutant	This study
Δ*FsCGBP*-2	*FsCGBP* gene deletion mutant	This study
CΔ*FsCGBP*	*FsCGBP* gene complementation strain	This study
GFP-*FsCGBP*	GFP-FsCGBP protein subcellular localization strain	This study
RNAi-*FsCGBP*	*FsCGBP* gene silencing strain	This study
Δ*FsHOG1*	*FsHOG1* gene deletion mutant	This study
Δ*FsMGV1*	*FsMGV1* gene deletion mutant	This study
Δ*FsGPMK1*	*FsGPMK1* gene deletion mutant	This study
Δ*FsCUT1*	*FsCUT1* gene deletion mutant	This study
Δ*FsCUT2*	*FsCUT2* gene deletion mutant	This study
Δ*FsCUT3*	*FsCUT3* gene deletion mutant	This study
Δ*FsCUT4*	*FsCUT4* gene deletion mutant	This study

Table S2 Plasmids used in this study

Plasmids	Description	Source
pUCHYG2	The plasmid contains hygromycin B phosphotransferase gene fragment that is used as a template for amplification.	The plasmid was modified by our laboratory
pCPXG418	The plasmid contains amino-glycoside 3 '-phosphotransferase (*APH (3) II*) gene, which can make *F.sacchari* resistant to geneticin (G418) after expression. The plasmid is used for gene complement.	The plasmid was modified by our laboratory
pCPXHY2	The plasmid contains hygromycin B phosphotransferase gene, which can make *F.sacchari* resistant to Hygromycin B after expression, and is used to construct RNA silencing expression vector of *F.sacchari*	The plasmid was modified by our laboratory
pCPXHY2-eGFP	The plasmid contains hygromycin B phosphotransferase gene and green fluorescent protein coding sequence, and is used for subcellular localization.	The plasmid was modified by our laboratory
pGBKT7	Used for yeast transactivation activity assays	Takara Biomedical Technology, China
pGADT7	The *FsCGBP* cDNA sequence was ligated to pGADT7 as the prey for yeast one-hybrid assays	Takara Biomedical Technology, China
pAbAi	Promoter of the target gene was ligated to the pAbAi as a bait	Miaoling Biotechnology, Wuhan, China
pGADT7-p53	Yeast one-hybrid assays positive control plasmid	Takara Biomedical Technology, China
pAbAi-p53	Yeast one-hybrid assays positive control plasmid	Miaoling Biotechnology, Wuhan, China
pCPXG418-*FsCGBP*	The plasmid was used for protoplast transformation of the deletion strain *ΔFsCGBP*, and the *FsCGBP* gene could be re-expressed after successful transformation	This study
pCPXHY2-RNAi-*FsCGBP*	The plasmid was used for protoplast transformation of FF001 strain, and the expression of *FsCGBP* gene could be inhibited after successful transformation	This study
pCPXHY2-eGFP-*FsCGBP*	The plasmid was used for protoplast transformation of FF001 strain, and *Fusarium* could translate eGFP-FsCGBP protein after successful transformation	This study

pGBKT7-*FsCGBP*	To verify whether FsCGBP is a transcription factor and detect its transcriptional activity	This study
pGADT7-*FsCGBP*	As the prey for yeast one-hybrid assays	This study
pAbAi-*FsCUT11*	As the bait for yeast one-hybrid assays	This study
pAbAi-*FsCUT12*	As the bait for yeast one-hybrid assays	This study
pAbAi-*FsCUT2*	As the bait for yeast one-hybrid assays	This study
pAbAi-*FsCUT31*	As the bait for yeast one-hybrid assays	This study
pAbAi-FsCUT32	As the bait for yeast one-hybrid assays	This study
pAbAi-*FsCUT4*	As the bait for yeast one-hybrid assays	This study
pAbAi-*FsHOG1*	As the bait for yeast one-hybrid assays	This study
pAbAi-*FsMGV1*	As the bait for yeast one-hybrid assays	This study
pAbAi-*FsGPMK1*	As the bait for yeast one-hybrid assays	This study

Table S3 Primers used in this study

Primers	Sequence（5'-3'）	Purpose	Notes
pGBKT7-*FsCGBP*-F	GCATATGGCCATGGAGGCCGATGGATTCCACAATGATGCCCC	Transcriptional activity analysis	The primers contained a homologous fragment of the pGBKT7 plasmid
pGBKT7-*FsCGBP*- R	GCGGCCGCTGCAGGTCGACGTTAGTCAGAGCGCTTGCGCT		
FsCGBP-F	CCCTTCTATTTCTACAACCCTGAGT	Validation of *FsCGBP* gene fragments	Shown as Fs-F/Fs-R in Figure S2 b,d
FsCGBP- R	GTCATTCCCGTGTTTGCTCCA		
FsCGBP-up-F	GGCGACGGATAGACTCGAAGTT	Knocking out the *FsCGBP*	Shown as up-F/R in Figure S2b
FsCGBP-up- *R*	ATATCATCTTCTGTCGACCTGCAGGCTAGCCACATATCCATTGCCTGGTAT		
FsCGBP-down-F	TCTTTCTAGAGGATCCCCGGGTACCGCGATTATCTTGACATACGCCCGATA	Knocking out the *FsCGBP*	Shown as down-F/R in Figure S2b
FsCGBP-down- *R*	AGACGACAGGGAGGCAGGTA		
hygF0	GAACCCGCTCGTCTGGCTAA	Gene knockouts were verified	Shown in Figure S2b
hygR0	CCGAGGGCAAAGGAATAGAGT		
C-FsCGBP-F	CATGCGTTAACAAGCTTGCGGCCGCCGCGCACTTTGTCTAGGGTC	Complementation strain	The primers contained a homologous fragment of the pCPXG418 plasmid
C-FsCGBP- R	AATATCATCTTCTGTCGACGAATTCCTGGACTTTTCTATTGCGACCTAGC		
G418-F0	AGGAAGGGCGAACTTAAGAAGG	Gene complementation were verified	Shown in Figure S2d
G418-R0	ACCACTGCACTAGGCCAC		
eGFP-FsCGBP-F	CTTTTAGAGGTACGCGGCCGCATGGATTCCACAATGATGCCCCAG	FsCGBP Subcellular Localization	The primers contained a homologous fragment of the pCPXHY2 plasmid
eGFP-FsCGBP- R	CTGCACCAGCTCCGCGGCCGCTGTCAGAGCGCTTGCGCT		
FsCGBP-RNAi-AL	CACTACTACTTTTAGGTACGCCACAAGCCCGCCATTCTGTT	Silencing strain	Amplification of interference fragment A
FsCGBP-RNAi- AR	GACAGTAACGCAAATTCAGTGTAGAGATTGACGAGGCTGTTGACGAA		
FsCGBP-RNAi-BL	ACCCCTCCAAAATCCCTTGTATGTTGATTGACGAGGCTGTTGACGAA	Silencing strain	Amplification of interference fragment B
FsCGBP-RNAi- BR	GTGGGAGATCAGGTCAGCATGCACAAGCCCGCCATTCTGTT		
Intron-F	AGAGGTACGCGGCCGCGTTAAGCTTCTACACTGAATTTGCGTTACTGTCT	Gene silencing were verified	Shown in Figure S2e
Intron- R	GATCAGGTCAGCATGCGTTGTTAACATACAAGGGATTTTGGAGGGGTG		

Primers	Sequence（5'-3'）	Purpose	Notes
pGADT7-*FsCGBP*-F	GGAGGCCAGTGAATTCATGGATTCCACAATGATGCCCCAG	Yeast one hybrid	The primers contained a homologous fragment of the pGADT7 plasmid
pGADT7-*FsCGBP*- R	CGAGCTCGATGGATCCGTCAGAGCGCTTGCGCT		
pAbAi-*HOG11-F*	AAGCTTGAATTCGAGCTTGGCGACCTTCTCACCATTT	Yeast one hybrid	
pAbAi-*HOG11- R*	GAGCACATGCCTCGAGGTCAATGCCCGTTGACAGGTAC		
pAbAi-MGV1-F	AAGCTTGAATTCGAGCTCAACCTGACGGCATTGAATCC	Yeast one hybrid	
pAbAi-MGV1- R	GAGCACATGCCTCGAGGAACGGCTCGGCCTCATCTA		
pAbAi-*GPMK13*-F	AAGCTTGAATTCGAGCTCTATACTCACCGTTGACGACGG	Yeast one hybrid	
pAbAi-*GPMK13*- R	GAGCACATGCCTCGAGGCAGCAGCTAAGGGTACCTACCA		
pAbAi-*CUT11*-F	AAGCTTGAATTCGAGCTCTTCGGATTTGCCTACCGTCA	Yeast one hybrid	
pAbAi-*CUT11*- R	GAGCACATGCCTCGAGGAGTTGGAAAGGCTTCCGAGT		
pAbAi-*CUT12*-F	AAGCTTGAATTCGAGCTGGGCATCAAGGTCCGTCT	Yeast one hybrid	
pAbAi-*CUT12*- R	GAGCACATGCCTCGAGGCGCGTACGATTAAATTCTCCCATT		
pAbAi-*CUT2*-F	AAGCTTGAATTCGAGCTAAATTCCCGTCAGCGAAGG	Yeast one hybrid	
pAbAi-*CUT2*- R	GAGCACATGCCTCGAGGTGTGAAACGGCGCCCTATG		
pAbAi-*CUT31*-F	AAGCTTGAATTCGAGCTAGGTGCCTTTTATACAAGCGCT	Yeast one hybrid	
pAbAi-*CUT31*- R	GAGCACATGCCTCGAGGCTTGCGTCTACTGCTATATCCACT		
pAbAi-*CUT32*-F	AAGCTTGAATTCGAGCTAATGTAAGGGATTCTGGGGCA	Yeast one hybrid	
pAbAi-*CUT32*- R	GAGCACATGCCTCGAGGGGTTCTCTTACTCGGGAAGCTTC		
pAbAi-*CUT4*-F	AAGCTTGAATTCGAGCTAGCGACACGCTCACCTAC	Yeast one hybrid	
pAbAi-*CUT4*- R	GAGCACATGCCTCGAGGTCGGAGGGGTTCAACATAGCC		
FsHOG1-up-F	CCTCCGTCCACATAATCCAATACC	Knocking out the *FsHOG1*	
FsHOG1-up- R	ATATCATCTTCTGTCGACCTGCAGGCAAGTAGTCAGTTTCCTGGCTGTAAA		
FsHOG1-down-F	TCTTTCTAGAGGATCCCCGGGTACCGATGTGGTTAATAACGGAGGAGGATG	Knocking out the *FsHOG1*	
FsHOG1-down- R	GTTGTCTCGGGTGAAATTCTATACG		
FsHOG1-F	GGCACCACATTCGAGATCAC	Validation of *FsHOG1* gene fragments	Shown as Fs-F/Fs-R in Figure S2 f

Primers	Sequence（5'-3'）	Purpose	Notes
FsHOG1- R	CTATTGTCCGTTAAATGGCTCCTCC		
FsMGV1-up-F	CCACTTGCTATGGAACACTGCA	Knocking out the *FsMGV1*	
FsMGV1-up- R	ATATCATCTTCTGTCGACCTGCAGGCTGTTCTGATATGATCTTGGATGCGG		
FsMGV1-down-F	TCTTTCTAGAGGATCCCCGGGTACCGGTATTCGCGTATGATAGGGGTCAG	Knocking out the *FsMGV1*	
FsMGV1-down- R	GTCATTGACTCTCAGCCCTGG		
FsMGV1-F	ATGTCGGACCTCCAAGGAC	Validation of *FsMGV1* gene fragments	Shown as Fs-F/Fs-R in Figure S2 f
FsMGV1- R	TATCTCCTAGAGGCATCCAGTCC		
FsGPMK13-up-F	ACGTAGGTAGTTGTTCCAGGTTC	Knocking out the *FsGPMK13*	
FsGPMK13-up-R	ATATCATCTTCTGTCGACCTGCAGGCCGGTGAGAAGGAGGGGAAAG		
FsGPMK13-down-F	TCTTTCTAGAGGATCCCCGGGTACCGGTATGATGACGAAGATGGGTGAGG	Knocking out the *FsGPMK13*	
FsGPMK13-down-R	ATACCGTCTTTTGTGCCATTGC		
FsGPMK13-F	ATGTCTCGATCGAACCCCC	Validation of *FsGPMK13* gene fragments	Shown as Fs-F/Fs-R in Figure S2 f
FsGPMK13- R	TGGTAGATCAACTGCTTCAGCTG		
FsCUT2-up-F	CATATTCCGCTATCGGAACGAC	Knocking out th*e* *FsCUT2*	
FsCUT2-up- R	ATATCATCTTCTGTCGACCTGCAGGCAGGCGAACAAGAATCGAAGACA		
FsCUT2-down-F	TCTTTCTAGAGGATCCCCGGGTACCGGTTGATGGAGGAAGGACTAGGC	Knocking out the *FsCUT2*	
FsCUT2-down-R	ACGAATTCGTGGCTGCCA		
FsCUT2-F	CTCTACTCTTCTCGCCGCCA	Validation of *FsCUT2 g*ene fragments	Shown as Fs-F/Fs-R in Figure S2 f
FsCUT2- R	CAGCATCAGCCTTCTGGATCA		
FsCUT3-up-F	AGCTTGGGACAGGTATTGGT	Knocking out the *FsCUT3*	
FsCUT3-up- R	ATATCATCTTCTGTCGACCTGCAGGCGGAGCGAGATTCGGAGACT		
FsCUT3-down-F	TCTTTCTAGAGGATCCCCGGGTACCGTGCCATTGTAGTTCTCCTCCG	Knocking out the *FsCUT3*	
FsCUT3-down- R	ACACGCACATTTCGACCAG		
FsCUT3-F	TGCAGCCATCCTTTCCTTCC	Validation of *FsCUT3* gene fragments	Shown as Fs-F/Fs-R in Figure S2 f
FsCUT3- R	AACCAGAAATTCCGGGCCT		

Primers	Sequence（5'-3'）	Purpose	Notes
FsCUT4-up-F	ACTCGTTATTTCCGTCCTCTCG	Knocking out the *FsCUT4*	
FsCUT4-up- R	ATATCATCTTCTGTCGACCTGCAGGCAGGACAGAGGATTTGCCGA		
FsCUT4-down-F	TCTTTCTAGAGGATCCCCGGGTACCGGTTGACAACTTGCCACTGACT	Knocking out the *FsCUT4*	
FsCUT4-down- R	TGACAAGGAGAGGCTACCCA		
FsCUT4-F	GCCATTGTCTATGCGGTCG	Validation of *FsCUT4* gene fragments	Shown as Fs-F/Fs-R in Figure S2 f
FsCUT4- R	CGTCACCTCGGTAGTTATGTTTTC		
qPCR-*Actin*-F	CGATTCTGGTGATGGTGTT	Reference genes for qRT-PCR	
qPCR-*Actin*- R	ACTCTTCCGTAGCAATGTC		
qPCR-*FsCGBP*-F	AGACTGCGGTGCTCGACTGG	qRT-PCR of *FsCGBP*	
qPCR-*FsCGBP*- R	ACGGAGCGGGCATAAGGC		
qPCR-HOG11-F	GCGCCCTCTTGAGAAGCAGTTCATC	qRT-PCR of *FsHOG11*	
qPCR-HOG11- R	CAGTCATCTGAGGGTCCTGGATTCG		
qPCR-MGV1-F	TGGAACCCCTAACGAAGAGACCCTC	qRT-PCR of FsMGV1	
qPCR-MGV1- R	GGGTCGAAGGCGAGCATCTTG		
qPCR-GPMK13-F	GCCAACGTGCTGCACCGAG	qRT-PCR of *FsGPMK13*	
qPCR-GPMK13- R	CGGGCGCACGGTACCATC		
qPCR-FsCUT1-F	TCTGTTCTCATCGCCCTCGTGC	qRT-PCR of *FsCUT1*	
qPCR-FsCUT1- R	AGCTGGGAATACCAGAGCCAAGG		
qPCR-FsCUT2-F	AACGACGCTAACCAGAAGTGTCCTG	qRT-PCR of *FsCUT2*	
qPCR-FsCUT2-/R	AGAACGACTCCAGCGATCTTGTCAC		
qPCR-FsCUT3-F	CAAGTTGTTGGAGCCGCCTTG	qRT-PCR of *FsCUT3*	
qPCR-FsCUT3- R	ACCAGATCGGTTGGCAGACAG		
qPCR-FsCUT4-F	CAAGATTGTCCTCGTCGGGCACAG		
qPCR-FsCUT4-R	GATAGGTACTGACCAGCGGCGATG	qRT-PCR of *FsCUT4*	
